# Design, Synthesis, and Biological Evaluation of a Novel [^18^F]-Labeled Arginine Derivative for Tumor Imaging

**DOI:** 10.3390/ph16101477

**Published:** 2023-10-16

**Authors:** Yong Huang, Chengze Li, Zhongjing Li, Yi Xie, Hualong Chen, Shengli Li, Ying Liang, Zehui Wu

**Affiliations:** 1 National Cancer Center, National Clinical Research Center for Cancer, Cancer Hospital & Shenzhen Hospital, Chinese Academy of Medical Sciences and Peking Union Medical College, Shenzhen 518116, China; 13260455651@163.com (Y.H.); lichengze1915@163.com (C.L.); lizhongjing321@163.com (Z.L.); 2 Beijing Institute of Brain Disorders, Laboratory of Brain Disorders, Ministry of Science and Technology, Collaborative Innovation Center for Brain Disorders, Capital Medical University, Beijing 100069, China; yixie@mail.ccmu.edu.cn (Y.X.); chenhl@ccmu.edu.cn (H.C.); 3 Department of Laboratory Animal Science, Capital Medical University, Beijing 100069, China; lilaoshi@ccmu.edu.cn

**Keywords:** Tracer 1, amino acid 2, (2S,4S)4-[^18^F]FPArg 3, arginine metabolism 4, positron emission tomography 5

## Abstract

To better diagnose and treat tumors related to arginine metabolism, (2S,4S)-2-amino-4-(4-(2-(fluoro-^18^F)ethoxy)benzyl)-5-guanidinopentanoic acid (**[^18^F]7**) was designed and prepared by introducing [^18^F]fluoroethoxy benzyl on carbon-4 of arginine. **[^18^F]7** and **7** were successfully prepared using synthesis methods similar to those used for (2S,4S)-4-[^18^F]FEBGln and (2S,4S)-4-FEBGln, respectively. In vitro experiments on cell transport mechanisms showed that **[^18^F]7** was similar to (2S,4S)4-[^18^F]FPArg and was transported into tumor cells by cationic amino acid transporters. However, **[^18^F]7** can also enter MCF-7 cells via ASC and ASC2 amino acid transporters. Further microPET-CT imaging showed that the initial uptake and retention properties of **[^18^F]7** in MCF-7 subcutaneous tumors were good (2.29 ± 0.09%ID/g at 2.5 min and 1.71 ± 0.09%ID/g at 60 min after administration), without significant defluorination in vivo. However, compared to (2S,4S)4-[^18^F]FPArg (3.06 ± 0.59%ID/g at 60 min after administration), **[^18^F]7** exhibited lower tumor uptake and higher nonspecific uptake. When further applied to U87MG imaging, **[^18^F]7** can quickly visualize brain gliomas (tumor-to-brain, 1.85 at 60 min after administration). Therefore, based on the above results, **[^18^F]7** will likely be applied for the diagnosis of arginine nutrition-deficient tumors and efficacy evaluations.

## 1. Introduction

Cancer remains a major disease that poses a threat to health (19.29 million new cancer cases worldwide in 2020), and the development of related diagnostic and treatment technologies has always been a research hotspot [1]. Radiopharmaceuticals are important tools for diagnosing and treating tumors [2]. Currently, radiopharmaceuticals can provide comprehensive diagnostic and treatment methods for tumors, including preoperative localization, companion diagnosis, treatment, and efficacy evaluation [3,4,5,6]. Therefore, a series of radiodiagnostic and therapeutic drugs targeting different targets have been developed [7,8,9,10,11], including [^177^Lu]PSMA-617 (Pluvicto) and [^68^Ga]Ga-PSMA-11, which target prostate-specific membrane antigen (PSMA) [12,13], and [^177^Lu]DOTATATE (Lutathera), which targets somatostatin receptor type 2 [14,15]; these drugs provide a new avenue for the diagnosis and treatment of tumors and greatly increase enthusiasm for the research and development of radiopharmaceuticals.

**Figure 1:**
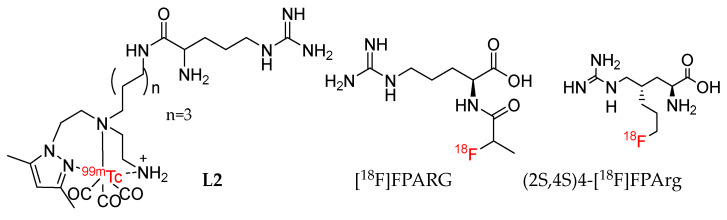
Chemical structures of **L2**, [^18^F]FPARG, and (2S,4S)4-[^18^F]FPArg.

Amino acids are the second largest source of energy for tumors. Different tumor cells require different types of amino acids. This process is regulated by the type and expression level of amino acid transporters [16,17]. Therefore, based on this difference in amino acid demand, a series of radiopharmaceuticals have been developed [16,18,19,20,21,22,23]. Arginine is an important substrate of cationic amino acid transport carriers and an important source of energy for a variety of tumors [24,25]. The main source of arginine in tumor cells is in vitro supply and self-synthesis. In vitro supply is the transport of arginine into cells through specific transporters [26]. Self-synthesis is the conversion of citrulline to arginine and is catalyzed by argininosuccinate synthase 1 (ASS1) and argininosuccinate lyase (ASL) [27,28]. However, some tumor subtypes (such as melanoma, hepatocellular carcinoma, and prostate cancer) cannot synthesize arginine because their ASS1 is not expressed [16,29]. These tumors must obtain exogenous arginine to maintain their metabolism [27,28]. Based on the negative correlation between the level of arginine uptake by tumor cells and the degree of ASS1 expression, the development of arginine metabolic molecular tracers can clarify the relationship between arginine metabolic level, ASS1 expression, tumor growth, and treatment time. Then, the tracers can provide effective imaging information to identify arginine-deficient tumors and monitor the efficacy of arginine deprivation therapy. Therefore, positron emission tomography (PET) and single photon emission computed tomography (SPECT) tracers targeting arginine metabolism have also been gradually reported (Figure 1). Correia et al. designed an **L2** SPECT tracer by introducing a bifunctional pyrazole complexing agent on the carboxyl group of arginine that can chelate with ^99m^Tc [30]. However, the tracer has not been further studied in vivo. Tang et al. designed and synthesized the tracer [^18^F]FPARG by introducing a 2-fluoropropionyl group on the amino group of arginine [31]. Micro PET imaging studies have shown that the tumor-to-muscle ratio of [^18^F]FPARG is approximately 2 at 30, 60, 90, and 120 min in NCI-H1299 tumor-bearing mice. Our group designed, synthesized, and obtained the tracer (2S,4S)4-[^18^F]FPArg by introducing a fluoropropyl group on carbon-4 of arginine [32]. The major difference between (2S,4S)4-[^18^F]FPArg and the previous two tracers is that the modified site was carbon-4 of arginine, so the characteristic groups of arginine (amino, carboxyl, and guanidine) were retained. Furthermore, the size of the modified group was smaller. Based on these reasons, (2S,4S)4-[^18^F]FPArg preserved the biological activity of arginine to the greatest extent. Breast cancer and glioma can be visualized with a high contrast through (2S,4S)4-[^18^F]FPArg [32,33]. However, this tracer has a low radiolabeling yield. To address this issue, herein, a novel arginine derivative was designed by introducing fluoroethoxy benzyl on carbon-4 of arginine; this was performed to increase the radiolabeling yield and attain a derivative with good tumor imaging abilities.

## 2. Results

### 2.1. Design and Synthesis of Compound 7

**Scheme 1:**
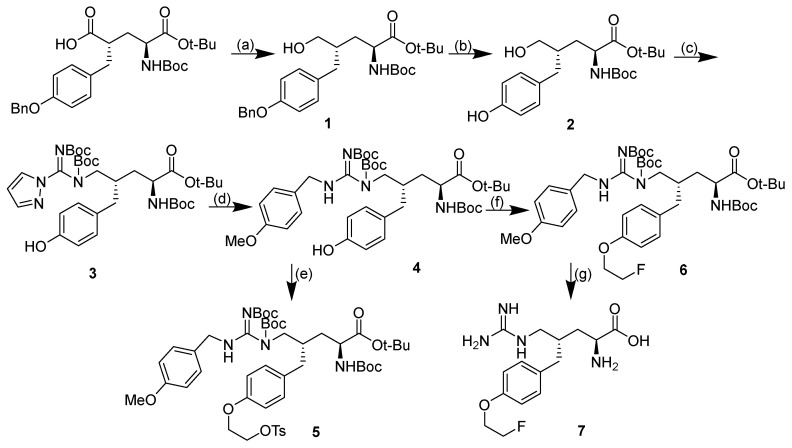
(**a**) Ethyl chloroformate, Et_3_N, NaBH_4_, THF, H_2_O, 0 °C—rt, 1 h; (**b**) Pd/C, H_2_, EtOH, rt, 2 h; (**c**) N,N’-di-Boc-1H-pyrazole-1-carboxamidine, triphenyl phosphine, diethyl azodicarboxylate, THF, 0 °C—rt, overnight; (**d**) 4-Methoxybenzylamine, N,N-diisopropylethylamine, ACN, 60 °C, 3 h; (**e**) 1,2-Bis(tosyloxy)ethane, K_2_CO_3_, DMF, rt, overnight; (**f**) 1-Bromo-2-fluoro-ethane, K_2_CO_3_, DMF, rt, overnight; (**g**) TFA, rt, 3 h.

In the synthesis route of (2S,4S)4-[^18^F]FPArg and (2S,4S)4-[^18^F]FEBGln [32,34], Compound **1** is a key intermediate that must be synthesized first. The introduction of guanidine groups is a key step in the synthesis route. Therefore, option 1 is to generate Compound **8** from Compound **1** through the Mitsunobu reaction and then reduce **8** to Compound **3** using Pd/C (Scheme S1 in Supplementary Materials). In option 2, Compound **1** is first reduced by Pd/C to generate Compound **2**, and then Compound **3** is obtained by the Mitsunobu reaction (Scheme 1). In Scheme 1, the hydroxyl group on the alkyl group of Compound **1** may react with its own phenolic hydroxyl group without generating Compound **3**. Therefore, Scheme S1 was carried out first. According to the previous method used to synthesize (2S,4S)4-[^18^F]FEBGln [34], we successfully obtained Compound **1** by reducing the carboxyl group. Compound **8** was successfully obtained by the Mitsunobu reaction (Scheme S1). However, after Compound **8** underwent Pd/C reduction, Compound **3** was not successfully obtained, and Compound **9** was produced. Thus, Scheme S1 was discarded, and Scheme 1 was performed. Compound **1** was reduced by Pd/C to obtain Compound **2**. Fortunately, Compound **3** was successfully obtained with a yield of 68.5% through the Mitsunobu reaction of Compound **2**. Compound **3** undergoes a nucleophilic substitution reaction with *p*-methoxybenzylamine to generate Compound **4** in a yield of 86.2%. Compound **4** was reacted with ethane-1,2-diyl bis(4-methylbenzenesulfonate) and 1-bromo-2-fluoroethane to obtain Compounds **5** and **6,** respectively. Compound **6** was hydrolyzed by TFA to obtain target Compound **7** in a yield of 38.9%. All compounds were characterized by NMR and HRMS.

### 2.2. Radiolabeling

**Scheme 2:**
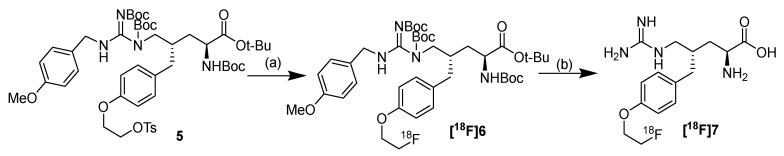
(**a**) 1 mL of 18-crown-6/KHCO_3_, [^18^F]F^−^, 100 °C, 15 min, tert-amyl alcohol and acetonitrile (9/1); (**b**) TFA, anisole, 60 °C, 5 min.

Our approach is to investigate the tumor imaging ability of **[^18^F]7** first and then optimize its radiolabeling conditions. Therefore, **[^18^F]7** was radiolabeled following the method used for (2S,4S)4-[^18^F]FEBGln to perform further imaging studies (Scheme 2). **[^18^F]7** was prepared with a radiochemical yield of 8% through a two-step reaction (without correction). **[^18^F]7** was characterized using different liquid phase conditions. **[^18^F]7** has a LogP of −1.27, which is higher than that of arginine due to the increased lipophilicity (CLogP=-3.52) after [^18^F]fluoroethoxy benzyl was introduced. An in vitro stability experiment found that **[^18^F]7** was stable in plasma and PBS within 2 h (Figure S2 in Supplementary Materials).

### 2.3. Cell Uptake Assays and Inhibition Studies

**Figure 2:**
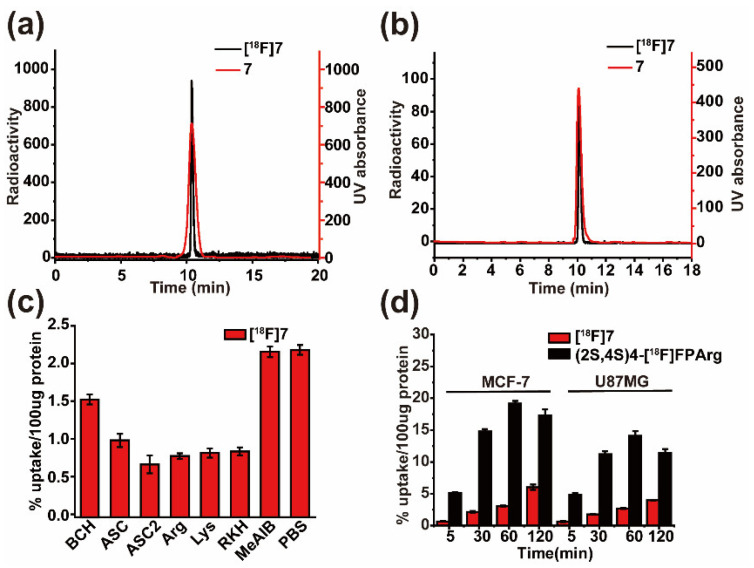
HPLC profiles of **7** and **[^18^F]7,** using Column: Agilent ZORBAX Eclipse XDB-C18, 250 mm × 9.4 mm, 5 μm, (**a**) and Astec CHIROBIOTIC^®^ TAG, 250 mm × 10 mm, 5 μm (**b**). (**c**) In vitro uptake of **[^18^F]7** in MCF-7 cells in the presence and absence of competitive inhibitors of amino acid transporter. MeAIB = 10 mM N-methyl α-aminoisobutyric acid (system A inhibitor); BCH = 10 mM 2-aminobicyclo[2.2.1]heptane-2-carboxylicacid (system L inhibitor); ASC = 3.3 mM each of L-Ala, L-Ser, L-Cys. ASC2 = 10 mM L-*γ*-glutamyl-*p*-nitroanilide; Arg = 10 mM arginine; Lys = 10 mM lysine; RKH = L-Arg, L-Lys, L-His mixture (3.3 mM of each amino acid). (**d**) In vitro cell uptakes of **[^18^F]7** and (2S,4S)4-[^18^F]FPArg.

The transport mechanisms of **[^18^F]7** were assessed through in vitro uptake assays with MCF-7 breast cancer cells in the absence and presence of amino acid transport inhibitors, and the results are depicted in Figure 2c. In the presence of MeAIB (an inhibitor of system A transporters), no inhibitory effect on uptake was observed, indicating that the system A transporter does not play a substantial role in the in vitro uptake of this tracer by MCF-7 cells. As expected, after cationic amino acid transporter substrates were added, such as arginine, lysine, and RKH, the uptake of **[^18^F]7** by MCF-7 cells was significantly reduced (approximately 64% reduction from the control), indicating that **[^18^F]7** is the substrate of the cationic amino acid transporter. In the presence of BCH, a competitive antagonist of system L, only 30% of the uptake of **[^18^F]7** was inhibited relative to the control, suggesting that only a portion of **[^18^F]7** uptake is mediated by system L. In the presence of a mixture of alanine, serine, and cysteine, which are used as inhibitors of a broad range of neutral amino acid transporters including system ASC, 54% of the uptake of **[^18^F]7** was inhibited. This result suggests that neutral amino acid transport systems partially mediate the uptake of this tracer by MCF-7 cells. When [^18^F]fluoroethoxy benzyl is introduced into the amino acid, the new tracer tends to be mediated into cells by ASC2, which is similar to the previously reported (2S,4S)4-[^18^F]FEBGln. In contrast to (2S,4S)4-[^18^F]FPArg [32], **[^18^F]7** enters MCF-7 cells primarily via cationic amino acid transporters. However, after the benzene ring is introduced, a large part of **[^18^F]7** can be mediated into the MCF-7 cell by ASC and ASC2; as a result, the uptake and retention of these two tracers in tumors are different. The uptake of (2S,4S)4-[^18^F]FPArg and **[^18^F]7** were further compared in different cell lines, and the uptake of (2S,4S)4-[^18^F]FPArg was significantly higher than that of **[^18^F]7** in MCF-7 and U87MG cells (Figure 2d). The introduction of 2-fluoroethoxy benzyl group not only changed the mechanism of cell uptake, but also decreased the amount of cell uptake, which was consistent with the results obtained for (2S,4S)4-[^18^F]FEBGln. Therefore, the structure of amino acids has a great influence on tumor uptake.

### 2.4. Small Animal PET-CT Imaging and Biodistribution in MCF-7 Subcutaneous Tumors

**Figure 3:**
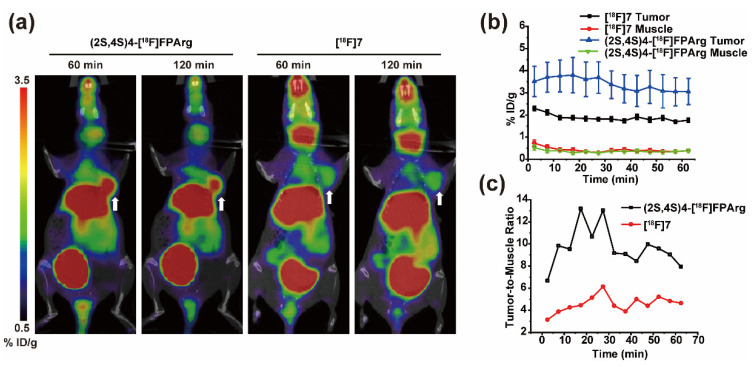
(**a**) The microPET-CT images of (2S,4S)4-[^18^F]FPArg and **[^18^F]7** in MCF-7 subcutaneous tumors, where white arrow indicates tumor; (**b**) Time-activity curves of (2S,4S)4-[^18^F]FPArg and **[^18^F]7** uptake in muscle and tumor; (**c**) The tumor-to-muscle ratio of (2S,4S)4-[^18^F]FPArg and **[^18^F]7** uptake in MCF-7 subcutaneous tumors.

The imaging ability of **[^18^F]7** for tumors was further investigated by microPET-CT imaging of MCF-7 subcutaneous tumors. As shown in Figure 3b, the uptake of **[^18^F]7** ranged from 2.29 ± 0.09%ID/g at 2.5 min to 1.71 ± 0.09%ID/g at 60 min after administration, which indicates that **[^18^F]7** has good tumor retention. Compared with (2S,4S)4-[^18^F]FPArg, **[^18^F]7** has a lower tumor uptake (Figure 3b). The muscle uptake of these two tracers was similar; therefore, the tumor-to-muscle ratio of (2S,4S)4-[^18^F]FPArg was higher than that of **[^18^F]7**(Figure 3c). Neither tracer exhibited significant in vivo defluorination (Figure 3a). From Figure S3, it can be seen that (2S,4S)4-[^18^F]FPArg and **[^18^F]7** are metabolized through the liver and kidney. The uptake of (2S,4S)4-[^18^F]FPArg in the liver and kidneys is close (Figure S3). When [^18^F]fluoroethoxy benzyl was introduced, the renal uptake of **[^18^F]7** was greatly increased. Correspondingly, **[^18^F]7** has a higher uptake in the bladder. Therefore, the introduction of benzene rings leads to changes in the metabolic pathway of the drug. The ratio of tumor uptake to liver or kidney uptake is low, so liver or kidney-related tumors cannot be accurately diagnosed with (2S,4S)4-[^18^F]FPArg and **[^18^F]7**. Although the imaging characteristics of **[^18^F]7** are not superior to those of (2S,4S)4-[^18^F]FPArg, **[^18^F]7** can accurately visualize MCF-7 subcutaneous tumors.

**Figure 4:**
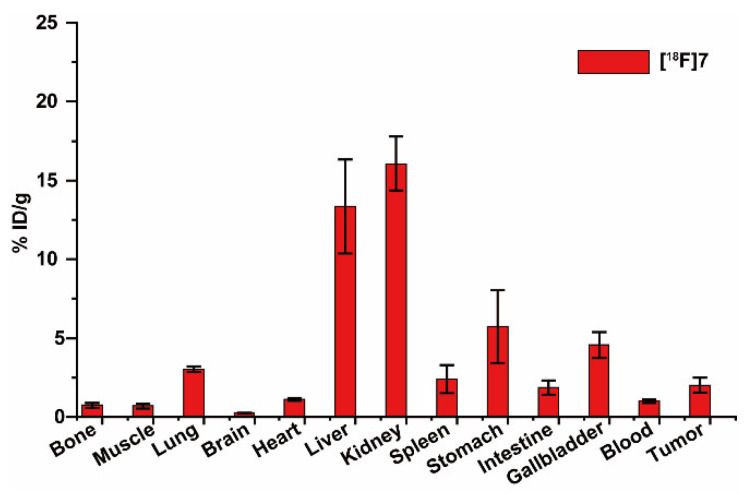
Biodistribution studies of **[^18^F]7** in mice bearing MCF-7 xenograft at 60 time points after intravenous injection (n = 4).

Further biodistribution experiments of MCF-7 subcutaneous tumor nude mice were performed to verify the results obtained by PET imaging (Figure 4). Consistent with PET imaging, **[^18^F]7** is mainly metabolized by the liver and kidney without obvious in vivo defluorination. **[^18^F]7** uptake is high in the gallbladder and stomach, and moderate in the spleen and lungs. As reported, the uptake of (2S,4S)4-[^18^F]FPArg is moderate in the intestine, and low uptake in the stomach, lungs, and spleen at 60 min post injection [32]. Therefore, compared to (2S,4S)4-[^18^F]FPArg, the uptake of **[^18^F]7** is low in the brain; therefore, **[^18^F]7** will likely be applied for glioma imaging in the same manner as other amino acid radiotracers.

### 2.5. Small Animal PET-CT Imaging in U87MG Intracranial Tumor Mouse Model

**Figure 5:**
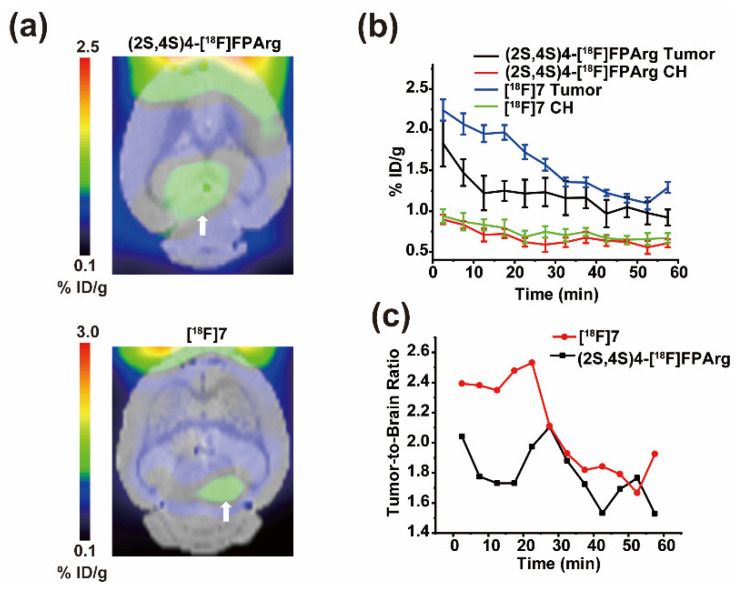
(**a**) The microPET-CT images of (2S,4S)4-[^18^F]FPArg and **[^18^F]7** in U87MG orthotopic glioma at 30 min post injection, where white arrow indicates tumor; (**b**) Time-activity curves of (2S,4S)4-[^18^F]FPArg and **[^18^F]7** uptake in contralateral hemisphere(CH)and tumor; (**c**) The tumor-to-brain ratio of (2S,4S)4-[^18^F]FPArg and **[^18^F]7** uptake in U87MG intracranial tumor.

Furthermore, U87MG intracranial tumors were used to verify whether **[^18^F]7** can visualize brain glioma. As shown in Figure 5b, **[^18^F]7** and (2S,4S)4-[^18^F]FPArg have high initial uptake in the tumor. However, within 30 min after administration, **[^18^F]7** showed better retention in tumors, and no significant difference was observed in brain uptake, so the contrast between **[^18^F]7** and tumor imaging within 30 min after administration is better than that of (2S,4S)4-[^18^F]FPArg (Figure 5a). Over time, the tumor and brain uptake of **[^18^F]7** and (2S,4S)4-[^18^F]FPArg were similar (Figure 5c), so imaging contrast was similar at 30 min after administration. Therefore, compared to (2S,4S)4-[^18^F]FPArg, **[^18^F]7** exhibits greater advantages in diagnosing glioma.

## 3. Discussion

Targeted arginine metabolism is crucial for the development of tumor diagnosis and treatment techniques, but no reports are available on related radioactive tracers. Therefore, our team developed a PET tracer (2S,4S)4-[^18^F]FPArg with a clear configuration targeting arginine metabolism for the first time [32]. However, the low yield and harsh condition of this tracer limit its clinical application. Two approaches were used to solve this problem. One was to optimize the labeling conditions of (2S,4S)4-[^18^F]FPArg. Another approach was to design novel tracers targeting arginine metabolism. Therefore, we attempted to design new tracers. Following the design concept of (2S,4S)4-[^18^F]FEBGln, **[^18^F]7** was obtained by introducing [^18^F]fluoroethoxy benzyl onto the skeleton of arginine.

By reducing the benzyl group first, then performing a Mitsunobu reaction and guanidine reaction, an important intermediate Compound **5** was successfully obtained. Compound **7** was obtained successfully by alkylation of Compound **5**. The labeling yields of (2S,4S)4-[^18^F]FPArg and **[^18^F]7** were low. The main reason is that the first step involved a low fluorination efficiency, whereas performing hydrolysis is more difficult in the subsequent steps with *p*-methoxybenzylamine. Therefore, the radiolabeling conditions for (2S,4S)4-[^18^F]FPArg and **[^18^F]7** still need to be further optimized by changing the protective group of the labeling precursor, such as replacing p-methoxybenzylamine with trimethoxybenzylamine (which is more easily hydrolyzed); using different phase transfer catalysts, such as tetrabutylammonium salts; and using different leaving groups, such as OMs or naphthalenesulfonyl groups.

Through cell uptake and inhibition experiments, it can be seen that **[^18^F]7** is the substrate of the cationic amino acid transporter. Unlike (2S,4S)4-[^18^F]FPArg, **[^18^F]7** is a substrate of ASC and ASC2. Further PET imaging of MCF-7 subcutaneous tumor-bearing mice showed that **[^18^F]7** could visualize tumors with a long retention time. However, compared to (2S,4S)4-[^18^F]FPArg, **[^18^F]7** had lower tumor uptake. Compared to (2S,4S)4-[^18^F]FEBGln with a similar structure[18], **[^18^F]7** has a longer tumor retention time in MCF-7 subcutaneous tumors. After [^18^F]fluoroethoxy benzyl was introduced, the newly designed glutamine derivative (2S,4S)4-[^18^F]FEBGln increased the contrast of tumor imaging compared to that of (2S,4R)4-[^18^F]FGln [18]. However, using the same method, the newly designed **[^18^F]7** failed to improve the tumor imaging contrast of (2S,4S)4-[^18^F]FPArg. Therefore, subtle differences in the structure of amino acids result in significant differences in tracer uptake and retention. Consistent with the advantages of radiolabeled amino acids in brain glioma imaging, **[^18^F]7** can also quickly visualize brain glioma. However, further studies are needed on the diagnosis and efficacy evaluation of **[^18^F]7** tumors in arginine deficiency.

Further comparison of reported tracers targeting arginine metabolism is shown in Table 1. The chemical synthesis steps of (2S,4S)4-FPArg and **7** are numerous, whereas the synthesis of **L2** and FPARG is relatively simple. The radiolabeling condition of compounds (2S,4S)4-[^18^F]FPArg, **[^18^F]7,** and [^18^F]FPARG is more complicated, whereas the radiolabeling conditions for **L2** are simpler. (2S,4S)4-[^18^F]FPArg, **[^18^F]7,** and **L2** all target cation amino acid transporter. (2S,4S)4-[^18^F]FPArg, **[^18^F]7,** and [^18^F]FPARG can visualize tumors. Only (2S,4S)4-[^18^F]FPArg and **[^18^F]7** have confirmed configurations and can visualize tumors. However, the radiolabeling yields of (2S,4S)4-[^18^F]FPArg and **[^18^F]7** are relatively low, which is not conducive to clinical application. Improving the labeling yield of (2S,4S)4-[^18^F]FPArg and **[^18^F]7** is the key to solving the clinical application of arginine tracers. Therefore, how to improve radiolabeling yield and target arginine metabolism is an important direction for developing next-generation tracers. On the premise of maintaining targeted arginine metabolism, the use of high radiolabeling yield isotope exchange methods, radioactive metal labeling methods, and click reactions are expected to develop new tracers.

## 4. Materials and Methods

All reagents used were commercial products and were used without further purification unless otherwise indicated. ^1^H NMR spectra were recorded at 300 MHz and ^13^C NMR spectra were measured at 75 MHz on a Bruker AV300 spectrometer at ambient temperature. Chemical shifts are reported in parts per million downfield from TMS (tetramethylsilane). Coupling constant in ^1^H NMR are expressed in Hertz. High-resolution mass spectrometry (HRMS) data were obtained with AB Sciex X500R QTof. Thin-layer chromatography (TLC) analyses were performed using Merck (Darmstadt, Germany) silica gel 60 F_254_ plates. Crude compounds generally were purified by flash column chromatography (FC) packed with Teledyne ISCO. All animal experiments were approved by the Animal Experiments and Experimental Animal Welfare Committee of Capital Medical University and carried out according to the guidelines of the Animal Welfare Act. The synthesis, radiolabeling, and biological evaluation procedures of **[^18^F]7** are shown in Table 2.

### 4.1. Synthesis

*tert-butyl (2S,4S)-4-(4-(benzyloxy)benzyl)-2-((tert-butoxycarbonyl)amino)-5 -hydroxypentanoate* (**1**)

(2S,4S)-2-(4-(benzyloxy)benzyl)-5-(tert-butoxy)-4-((tert-butoxycarbonyl)amino)-5-oxopentanoic acid (1 g, 2.12 mmol) was dissolved in 25 mL THF in a 50 mL round bottom flask and the solution was cooled to 0 °C. To this solution, Et_3_N (0.22 mL, 2.12 mmol) and ethyl chloroformate (0.26 mL, 2.2 mmol) were added dropwise. After stirring at 0 °C for 30 min, the reaction mixture was filtered off. To a mixture of NaBH_4_ (0.17 g, 4.24 mmol) with 2 mL H_2_O in a 100 mL round bottom flask cooled with an ice bath the above filtrate was added slowly. The mixture was stirred at room temperature for a further 1 h and was then acidified with 1 M HCl until the pH = 7 under cooling with an ice bath. The organic phase was collected and the water phase was extracted with ethyl acetate (20 mL × 3). The organic phases were combined, washed with saturated NaHCO_3_ (20 mL) and brine (20 mL), and dried with MgSO_4_. The filtrate was evaporated in vacuo and the residue was purified by FC (ethyl acetate/hexane 40/60) to give oil **1** (0.65 g, 63.7%). ^1^H NMR (300 MHz, CDCl3) δ 7.44–7.28(m, 5H), 7.11 (d, J = 8.4 Hz, 2H), 6.91 (d, J = 8.5 Hz, 2H), 5.25 (s, 1H), 5.05 (s, 2H), 4.29 (s, 1H), 3.77–3.74 (d, J = 10.0 Hz, 1H), 3.51–3.48 (m, 1H), 2.64 (s, 2H), 1.94 (s, 1H), 1.69 (s, 1H), 1.47–1.44 (m, 18H). ^13^C NMR (75 MHz, CDCl_3_) δ 172.85, 157.66, 155.21, 136.98, 130.33, 129.91, 129.62, 128.56, 127.94, 127.42, 115.13, 80.24, 77.40, 77.18, 76.98, 76.56, 70.47, 70.09, 69.18, 47.58, 37.57, 34.01, 32.99, 31.90, 31.21, 28.30, 27.93. HRMS calcd for C28H40NO6+ 486.2850 [M+H]^+^, found, 486.2851.

*tert-butyl (2S,4S)-2-((tert-butoxycarbonyl)amino)-5-hydroxy-4-(4-hydroxybenzyl) pentanoate* (**2**)

A mixture of 1 (0.5 g, 1 mmol) and 10% Pd/C (0.1 g) in absolute EtOH (20 mL) was stirred under H_2_ for 3 h. This mixture was then filtered and the filtrate was concentrated under vacuum to give a white solid **2** (0.4 g, 100%).^1^H NMR (300 MHz, CDCl_3_) δ 7.01 (d, J = 8.2 Hz, 2H), 6.74 (d, J = 8.3 Hz, 2H), 5.25 (s, 1H), 4.26 (s, 1H), 3.75–3.72 (m, 1H), 3.50 (s, 1H), 2.61–2.57 (m, 2H), 1.93 (s, 1H), 1.71 (t, J = 6.7 Hz, 1H), 1.44 (d, J = 7.1 Hz, 9H). HRMS calcd for: C21H34NO6+ 396.2381 [M+H]+, found, 396.2383.

*tert-butyl (2S,4S)-5-((Z)-N,N′-bis(tert-butoxycarbonyl)-1H-pyrazole-1- carboximidamido)-2-((tert-butoxycarbonyl)amino)-4-(4-hydroxybenzyl)pentanoate* (**3**)

To a solution of N,N′-Di-Boc-1H-pyrazole-1-carboxamidine(0.31 g, 1 mmol), **2** (0.4 g, 1 mmol) and triphenylphosphine (0.52 g, 2 mmol) at 0 °C in anhydrous THF, diethyl azodicarboxylate (0.34 mL, 2 mmol) was added dropwise; after 10 min, the reaction was warmed at room temperature and stirred overnight. The solvent was removed under vacuum, and purified by FC (ethyl acetate/hexane 20/80) to obtain colorless oil **3** (0.52 g, 68.5%) ^1^H NMR (300 MHz, CDCl_3_) δ 7.95 (s, 1H), 7.79–7.63 (m, 1H), 7.61–7.47 (m, 1H), 7.09–6.89 (m, 2H), 6.75–6.69 (m, 2H), 6.57 (s, 1H), 5.20–5.08 (m, 1H), 4.21–3.85 (m, 2H), 3.68–3.63 (m, 1H), 2.72–2.07 (m, 4H), 1.54–1.25 (m, 36H). ^13^C NMR (75 MHz, CDCl_3_) δ 172.53, 167.77, 156.43, 154.81, 132.26, 130.92, 130.28, 130.17, 128.82, 115.30, 82.44, 82.19, 81.90, 81.13, 78.68, 70.25, 65.39, 59.35, 52.58, 30.53, 28.36, 28.30, 27.95, 27.85, 27.65, 21.91, 19.14, 13.68. HRMS calcd for: C35H54N5O9+ 688.3916 [M+H]^+^, found, 688.3919.

*tert-butyl (2S,4S)-5-((E)-1,2-bis(tert-butoxycarbonyl)-3-(4-methoxybenzyl)guanidino) -2-((tert-butoxycarbonyl)amino)-4-(4-hydroxybenzyl)pentanoate* (***4***)

To a solution of **3** (0.5 g, 0.72 mmol) at room temperature in anhydrous acetonitrile, *p*-methoxybenzylamine (0.14 mg, 0.72 mmol) was added dropwise, and after 10 min, the reaction was warmed at 60 °C for 3 h. The solvent was removed under vacuum, and purified by FC (ethyl acetate/hexane 30/70) to obtain colorless oil **4** (0.47 g, 86.2%).^1^H NMR (300 MHz, CDCl_3_) δ 7.19 (d, *J* = 8.2 Hz, 2H), 6.95 (d, *J* = 7.6 Hz, 2H), 6.87 (d, *J* = 8.2 Hz, 2H), 6.72 (d, *J* = 8.2 Hz, 2H), 5.29 (s, 1H), 4.34 (s, 2H), 4.16–4.01 (m, 1H), 3.79 (s, 3H), 3.72–3.46 (m, 2H), 2.77–2.71 (m, 1H), 2.43 (s, 1H), 2.12 (s, 1H), 1.53 (m, 37H). ^13^C NMR (75 MHz, CDCl_3_) δ 172.34, 159.33, 155.81, 154.97, 129.96, 129.30, 115.35, 114.32, 82.53, 81.59, 79.67, 60.39, 55.26, 53.41, 52.12, 51.21, 47.27, 36.94, 34.63, 28.04, 20.90. HRMS calcd for: C40H61N4O10+, 757.4382 [M+H]^+^; found, 757.4384.

*tert-butyl (2S,4S)-5-((E)-1,2-bis(tert-butoxycarbonyl)-3-(4-methoxybenzyl)guanidino) -2-((tert-butoxycarbonyl)amino)-4-(4-(2-(tosyloxy)ethoxy)benzyl)pentanoate* (***5***)

A solution of **4** (0.15 g, 0.19 mmol) and K_2_CO_3_ (0.14 g, 1 mmol) was added in 15 mL DMF was stirred at room temperature for 0.5 h, and 1,2-Bis(tosyloxy)ethane (0.3 g, 0.75 mmol) was added. The reaction was stirred at room temperature overnight. The reaction was extracted by ethyl acetate (50 mL), washed by saturation NaCl, dried by Na_2_SO_4_, and purified by FC (ethyl acetate/hexane 30/70) to give **5** (0.13 g, 67.8%) as an oil. ^1^H NMR (300 MHz, CDCl_3_) δ 7.79 (d, *J* = 8.2 Hz, 2H), 7.32 (d, *J* = 8.0 Hz, 2H), 7.18 (d, *J* = 8.3 Hz, 2H), 7.05 (d, *J* = 7.9 Hz, 2H), 6.86 (d, *J* = 8.5 Hz, 2H), 6.65 (d, *J* = 8.2 Hz, 2H), 5.09 (s, 1H), 4.32 (s, 4H), 4.06 (d, *J* = 4.9 Hz, 2H), 3.74 (s, 3H), 3.61–3.52 (m, 2H), 2.79 (d, *J* = 4.8 Hz, 2H), 2.42 (s, 4H), 2.10 (s, 1H), 1.61 (d, *J* = 5.1 Hz, 1H), 1.45–1.38 (m, 36H). ^13^C NMR (75 MHz, CDCl_3_) δ 172.15, 159.35, 156.42, 155.70, 153.40, 144.90, 132.91, 132.14, 130.40, 129.83, 129.10, 127.96, 114.38, 114.30, 82.57, 81.69, 79.53, 68.16, 65.43, 55.25, 52.01, 51.04, 47.23, 36.66, 28.32, 28.18, 28.05, 27.93, 21.59. HRMS calcd for C49H71N4O13S+ 955.4733 [M+H]^+^, found, 955.4736.

*tert-butyl (2S,4S)-5-((E)-1,2-bis(tert-butoxycarbonyl)-3-(4-methoxybenzyl)guanidino) -2-((tert-butoxycarbonyl)amino)-4-(4-(2-fluoroethoxy)benzyl)pentanoate* (***6***)

A solution of **4** (0.15 g, 0.19 mmol) and K_2_CO_3_ (70 mg, 0.5 mmol) was added in 10 mL DMF was stirred at room temperature for 0.5 h, and 1-Bromo-2-fluoro-ethane (47 mg, 0.38 mmol) was added. The reaction was stirred at room temperature overnight. The reaction was extracted by ethyl acetate (50 mL), washed by saturation NaCl, dried by Na_2_SO_4_, and purified by FC (DCM/Methanol = 10/1) to give **6** (0.13 g, 81.1%) as an oil. ^1^H NMR (300 MHz, CDCl_3_) δ 7.22 (d, *J* = 8.3 Hz, 2H), 7.12 (d, *J* = 7.9 Hz, 2H), 6.89 (d, *J* = 8.5 Hz, 2H), 6.81 (d, *J* = 8.1 Hz, 2H), 5.01 (s, 1H), 4.82 (s, 1H), 4.75–4.62 (m, 1H), 4.35 (s, 2H), 4.28–4.17 (m, 1H), 4.12 (s, 1H), 3.81 (s, 3H), 3.74–3.52 (m, 2H), 2.83 (s, 1H), 2.49 (s, 1H), 2.13 (s, 1H), 1.63–1.35 (m, 37H). HRMS calcd for: C42H64FN4O10+, 803.4601 [M+H]^+^, found, 803.4606.

*(2S,4S)-2-amino-4-(4-(2-fluoroethoxy)benzyl)-5-guanidinopentanoic acid* (***7***)

A solution of compound **6** (0.1 g, 0.12 mmol) was added in 3 mL TFA, and the reaction was stirred at room temperature for 2 h. TFA was removed, ether (10 mL) was added, and solid precipitation was collected. The solid was dissolved in 1 mL H_2_O and purified by HPLC to give white solid **7**(40 mg, 38.9%). ^1^H NMR (300 MHz, CD_3_OD) δ 7.12 (d, *J* = 8.5 Hz, 2H), 6.89 (d, *J* = 8.5 Hz, 2H), 4.83–4.72 (m, 1H), 4.72–4.56 (m, 1H), 4.30–4.19 (m, 1H), 4.18–4.07 (m, 1H), 3.74–3.71 (m, 1H), 3.55–3.49 (m, 1H), 3.05–2.98 (m, 1H), 2.88–2.82 (m, 1H), 2.55–2.37 (m, 1H), 2.28 (s, 1H), 1.83–1.75 (m, 1H), 1.67–1.57 (m, 1H). ^13^C NMR (75 MHz, DMSO-*d6*) δ 171.49, 159.46, 158.87, 157.95, 157.06, 131.45, 130.67, 114.77, 106.20, 83.72, 81.35, 67.60, 67.10, 50.61, 48.88, 43.53, 42.56, 36.33, 36.03, 35.59, 32.78. HRMS calcd for: C15H24FN4O3+, 327.1827 [M+H]^+^, found, 327.1829.

### 4.2. Radiolabeling

[^18^F]Fluoride was purchased from the company of DONGCHENG AMS (Guangdong) PHARMACEUTICAL with an HM-20 medical cyclotron (Sumitomo, Kyoto, Japan) as an [^18^O] enriched aqueous solution of [^18^F]fluoride. Solid-phase extraction (SPE) cartridges such as Sep-Pak QMA Light and Oasis HLB cartridges were purchased from Waters (Milford, MA). High-performance liquid chromatography (HPLC) was performed on Agilent 1260 Infinity II system with different HPLC columns. The radiosynthesis condition of (2S,4S)-[^18^F]FPArg was conducted following our previous method [33].

The radiosynthesis condition of **[^18^F]7** was conducted following (2S,4S)4-[^18^F]FEBGln’s radiolabeling method. The typical experimental steps are as follows. An activated Sep-Pak Light QMA Carb was loaded with 1050 MBq (28.4 mCi) of [^18^F]fluoride and eluted with 1 mL of 18-crown-6/KHCO_3_ (480 mg of 18-crown-6 in 18.6 mL of ACN/90 mg of KHCO_3_ in 3.4 mL of water). The solution was blown with argon until dry and dried twice azeotropically with 1 mL of acetonitrile at 100 °C under a flow of argon. The dried [^18^F]fluoride was cooled in an ice bath and 5 mg of tosylate precursor **5** was dissolved in 1 mL of tert-amyl alcohol and acetonitrile (9/1) and added to the dried [^18^F] fluoride. The mixture was heated for 15 min at 100 °C in an oil bath. The mixture was then cooled in an ice bath and added to 8 mL of water/1 mL of acetonitrile. The mixture was loaded on an activated Oasis HLB 3 cm^3^ cartridge, pushed through, and washed with 3 mL of water/acetonitrile (7/3) and 2 mL of water/acetonitrile (1/1) sequentially. The desired radiolabeled compound was eluted with 1 mL of ethanol. The radiochemical purity of the intermediate was assessed by coinjection of the nonradioactive standard **6**, onto a semi-preparation column ((9.4 mm × 250 mm, 5 μm, ZORBAX Eclipse XDB-C18, Agilent) using a solution of 0.1% formic acid) = A, B = ACN, 0–2 min 90% A/10% B, 2–15 min 90% A/10% B→20% A/80% B, 15–25 min 20% A/80%) as mobile phase with a flow rate of 3 mL/min and λ = 254 nm. **[^18^F]6** had retention times of 19.87 min (Figure S1).

The ethanol solution was blown until dry. TFA (1 mL) was added and heated for 5 min at 60 °C in a capped 10 mL vial. TFA was removed under argon while still warm. The reaction tube was then cooled in an ice bath. An amount of 1 mL of 10% ethanol physiological saline solution was slowly added into the mixture, vortexed and mixed, filtered by a sterile membrane, and yielded the desired radioactive **[^18^F]7** (pH = 5~7). 

The radiochemical and stereochemical purities were determined by two different HPLC systems. System 1. Column: ZORBAX Eclipse XDB-C18, Agilent, 9.4 mm × 250 mm, 5 μm), mobile phase (gradient elution): A = 0.1% formic acid, B = ACN, 3 mL/min, 0–2 min 90% A/10% B, 2–15 min 90% A/10% B→20% A/80% B, 15–25 min 20% A/80% B) at 30 °C. The retention times of **[^18^F]7** are 10.12 min (Figure 2a). System 2. Column: Astec CHIROBIOTIC^®^ TAG, 25 cm × 10 mm, 5 μm, mobile phase (isocratic): H_2_O/methanol 40/60, 2 mL/min, column temperature at 30 °C. The retention times of **[^18^F]7** are 10.08 min (Figure 2b).

### 4.3. Partition Coefficient and in Vivo Stability

Partition coefficient (Log P) and in vivo stability were following our previous reported methods [18].

### 4.4. In Vitro Cell Uptake Studies

To characterize the transport of **[^18^F]7**, competitive inhibition studies were conducted using the MCF-7 cell line (Figure 2c). The tracer was incubated at 37 °C for 30 min in MCF-7 cells. The cells were processed as described above. Various inhibitors were then added to the cells in PBS solution at a concentration of 10 mM. Selected inhibitors included synthetic amino acid transport inhibitors, such as N-methyl-α aminoisobutyric acid (MeAIB) for system A, 2-amino-bicylo[2.2.1] heptane-2-carboxylic acid (BCH) for system L, 3.3 mM each of L-Ala, L-Ser, L-Cys for system ASC, and L-γ-Glutamyl-pnitroanilide (GPNA) for system ASCT2, L-Arg, L-Lys, and RHK for system CAT. The data was compared in reference to the uptake of **[^18^F]7**, without any inhibitor in PBS solution at pH 7.4. The data were normalized as a percentage uptake of initial dose (ID) relative to % uptake/100 μg of protein.

MCF-7 cells and U87MG were cultured in RPMI 1640 (SIGMA, St. Louis, MO, USA) supplemented with 10% fetal bovine serum (YHSM) and 1% penicillin/streptomycin (Gibco, Billings, MT, USA). The cells were maintained in T-75 culture flasks under humidified incubator conditions (37 °C, 5% CO_2_) and were routinely passaged at the confluence. Tumor cells were plated (2.0 × 10^5^ cells/well) 24 h in the media prior to ligand incubation. On the day of the experiment, the culture media was aspirated and the cells were washed three times with warm PBS (containing 0.90 mM of Ca^2+^ and 1.05 mM of Mg^2+^). (2S,4S)-[^18^F]FPArg or [^18^F]**7** (37 kBq/mL/well) were mixed in PBS (with Ca^2+^ and Mg^2+^) solution and then added to each well. The cells were incubated at 37 °C for 5, 30, 60, and 120 min. At the end of the incubation period, the PBS solution containing the ligands was aspirated and the cells were washed three times with 1 mL of ice-cold PBS (without Ca^2+^ and Mg^2+^). After washing with ice-cold PBS, 350 μL of 1M NaOH was used to lyse the cells. The lysed cells were collected onto filter paper and counted together with samples of the incubation dose using a gamma counter. A total of 100 μL of the cell lysate was used to determine the protein concentration (Modified Lowry Protein Assay). The data were normalized as percentage uptake of initial dose (ID) relative to %uptake/100 μg of protein.

### 4.5. MicroPET-CT Imaging

Nude mice (female, weight, 12–16 g) bearing MCF-7 xenografts and U87MG intracranial tumors were purchased from Guangdong GemPharmatech Co., Ltd. (Nanjing, China). Data were recorded on a Madiclab PSA146 PET/CT/FMT instrument. Dynamic small animal PET-CT imaging studies were conducted with **[^18^F]7** or (2S,4S)4-[^18^F]FPArg, similar to that reported previously [35]. All scans were performed on a dedicated animal PET scanner. Nude mice with MCF-7 xenografts tumors were used for the imaging studies. A total of 8-11 MBq **[^18^F]7** of activity was injected intravenously via the lateral tail vein. For nude mice bearing MCF-7 xenografts tumors, dynamic PET images were collected for 1 h after administration of 8-11 MBq **[^18^F]7** or (2S,4S)4-[^18^F]FPArg(Figure 3). For nude mice bearing U87MG intracranial tumors, dynamic PET images were collected for 1 h after administration of 8–11 MBq **[^18^F]7** or (2S,4S)4-[^18^F]FPArg (Figure 5). All animals were sedated with isoflurane anesthesia (2–3%, 1 L/min oxygen) and were then placed on a heating pad to maintain body temperature throughout the procedure. The animals were visually monitored for breathing and any other signs of distress throughout the entire imaging period. The data acquisition began after an intravenous injection of the tracer. Micro-PET/CT images were analyzed by using Pmod software (version 4.0, PMOD Technologies Ltd., Zurich, Switzerland). Each microPET image was manually co-registered to the Mirrione T2 mouse brain template by using rigid body transformation [36]. Then, the resulting transformation parameters were applied to the corresponding micro-PET image. Four volumes of interest (VOIs) were selected from the Mirrione atlas. TACs were extracted from all the VOIs and performed as the percentage injection dose per cubic centimeter (%ID/g). The TAC of normalized to pick value was also calculated.

### 4.6. Biodistribution

Biodistribution was following our previous reported methods [18].

## 5. Conclusions

**[^18^F]7** was designed by introducing [^18^F]fluoroethoxy benzyl into carbon-4 of arginine. By retrosynthesis, 7 was successfully prepared. **[^18^F]7** was successfully radiolabeled with 8% radiochemical yield (without correction) following the radiolabeling method used for (2S,4S)-4-[^18^F]FEBGln. In vitro cell uptake inhibition experiments showed that **[^18^F]7** can be taken up by cells through the mediation of cationic, ASC, and ASC2 amino acid transporters. [^18^F]7 can visualize MCF-7 subcutaneous tumors with a long tumor retention time (2.29 ± 0.09%ID/g at 2.5 min and 1.71 ± 0.09%ID/g at 60 min after administration), but the imaging contrast (**[^18^F]7** and (2S,4S)4-[^18^F]FPArg tumor-to-muscle ratio is approximately 5 and 10, respectively, within 60 min after administration) and specific uptake are lower than those of (2S,4S)4-[^18^F]FPArg. In terms of glioma imaging, **[^18^F]7** has a certain advantage over (2S,4S)4-[^18^F]FPArg. Therefore, [^18^F]7 will likely be used in the diagnosis of arginine nutrition-deficient tumors and efficacy evaluations.

## Figures and Tables

**Table 1:** Comparison of targeted arginine metabolism tracers.

Tracer	Organic Synthesis	Radiolabeling	Evaluation
(2S,4S)4-[^18^F]F PArg [32,33]	10 step, Configuration determination	2 step, yield < 6%	Targeting CAT transporters; PET imaging, MCF-7 (T/M = 6) and U87MG (T/M = 6) subcutaneous tumor, U87MG (T/B =1.5) and HS683 (T/B = 6.5) intracranial tumor, at 60 min p.i.
[^18^F]7	10 step, Configuration determination	2 step, yield = 8%	Targeting CAT, ASC, ASC2 transporters, PET imaging, MCF-7 Tumor (T/M = 4), intracranial tumor (T/B = 2) at 60 min p.i.
L2 [30]	3 step, Configuration determination	1 step, yield = 100%	Targeting y^+^ transporters, no PET Imaging
[^18^F]FPARG [31]	4 step, Configuration undetermined	5 step, yield = 15 ± 3%	No testing of transport mechanism, PET imaging, NCI-H460 Tumor (T/M = 2) at 60 min p.i.

CAT: Cationic amino acid transporter, p.i.: post injection, T/M: Tumor/Muscle, T/B: Tumor/Brain.

**Table 2:** Synthesis and Evaluation of [^18^F]7.

Synthesis	Radiolabeling	Evaluation		
		In Vitro	In Vivo	
**Compound** 1–7	Two-step radiolabeling method follow	Partition Coefficient	PET-CT imaging compared with (2S,4S)4-[^18^F]FPArg	MCF-7 subcutaneous tumor
		Stability in PBS and Plasma		U87MG intracranial tumors
		Cell uptake	Biodistribution	

## Data Availability

Data are contained within the article or Supplementary Materials.

## Supplementary Materials

The following supporting information can be downloaded at: https://www.mdpi.com/article/10.3390/ph16101477/s1, Figure S1. HPLC profiles of “cold” **6** and **[^18^F]6**, Figure S2. The stability in PBS and plasma of **[^18^F]7**, Figure S3. Time-activity curves of (2S,4S)4-[^18^F]FPArg and **[^18^F]7** uptake in U87MG tumor-bearing nude mice liver and kidney.

## Abbreviations

ACN, Acetonitrile; Et_3_N, triethylamine; PBS, phosphate buffer saline; THF, tetrahydrofuran; TFA, trifluoroacetic acid; HPLC, high-performance liquid chromatography; HRMS, high-resolution mass spectrometry; PET, positron emission tomography.
