# Supplementary material for: Design, Synthesis, and Biological Evaluation of a Novel [18F]-Labeled Arginine Derivative for Tumor Imaging
Source: Pharmaceuticals (Basel). 2023 Oct 16;16(10):1477. doi: 10.3390/ph16101477 (PMC10610273; doi:10.3390/ph16101477)
Supplement: Supplementary file 1 [file pharmaceuticals-16-01477-s001.zip › pharmaceuticals-2621534-supplementary.pdf]

Supplemental Information

Design, synthesis and biological evaluation of a novel [^{18}F]-labeled arginine derivative for tumor imaging

1. Synthesis. 2
2. Radiolabeling and *in vitro* stability 2
3. MicroPET-CT imaging. 3
4. NMR of compound 4

1. Synthesis.

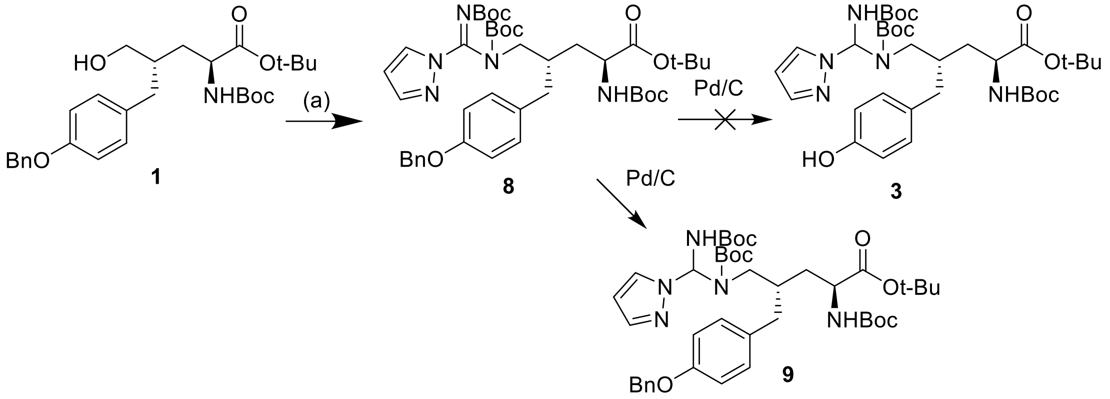

Scheme S1. The initial route for synthesizing compound **3**. (a) N,N’-Di-Boc-1H-pyrazole-1-carboxamidine, triphenyl phosphine, diethyl azodicarboxylate, THF, 0 °C - rt, overnight.

2. Radiolabeling and *in vitro* stability

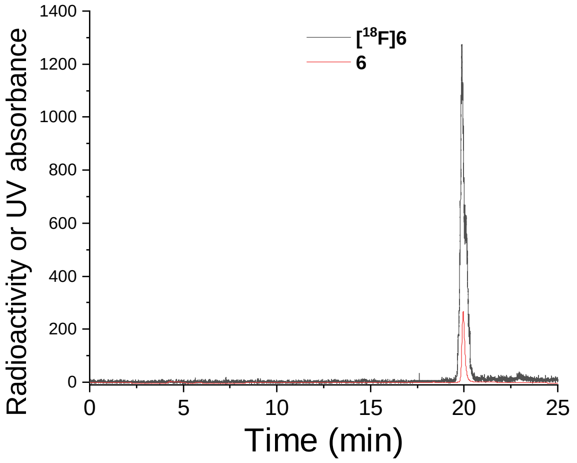

Figure S1. HPLC profiles of “cold” **6** and **[^{18}F]6.**

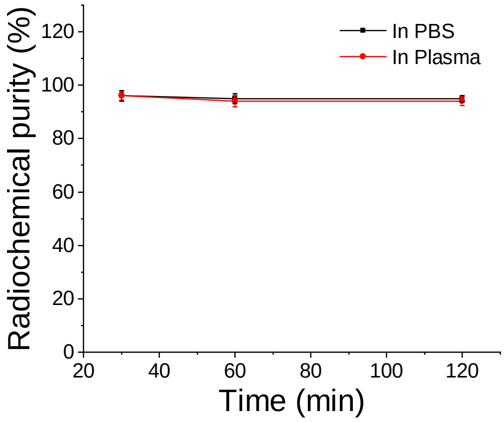

Figure S2. The stability in PBS and plasma of **[^{18}F]7.**

3. MicroPET-CT imaging.

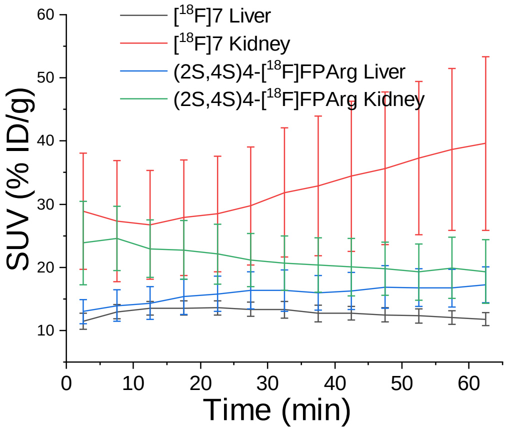

Figure S3. Time-activity curves of (2S,4S)4-[^{18}F]FPArg and **[^{18}F]7** uptake in liver and kidney of U87MG tumor-bearing nude mice.

4. NMR of compound

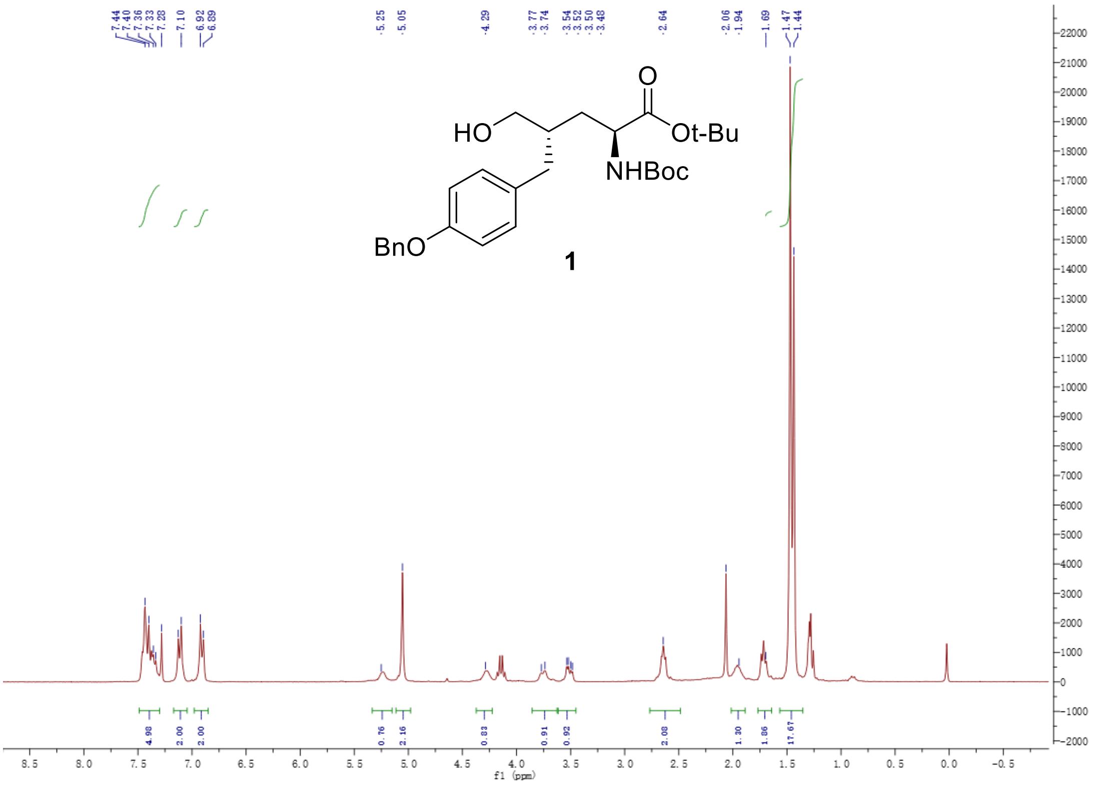

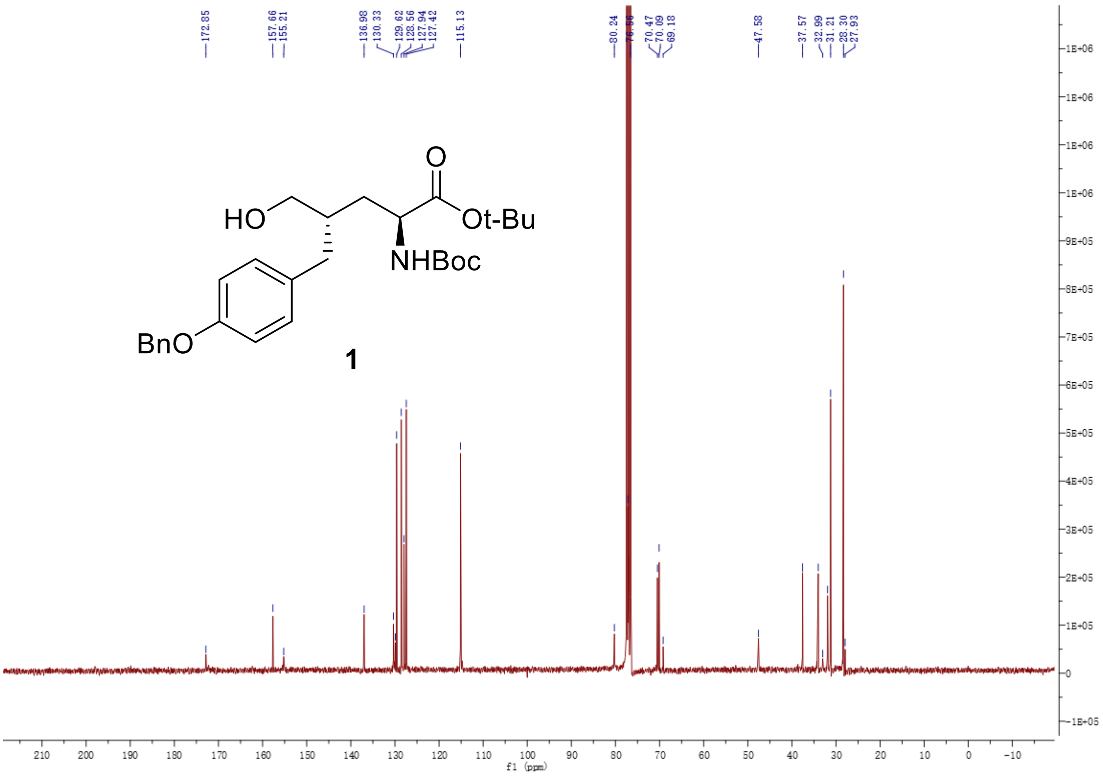

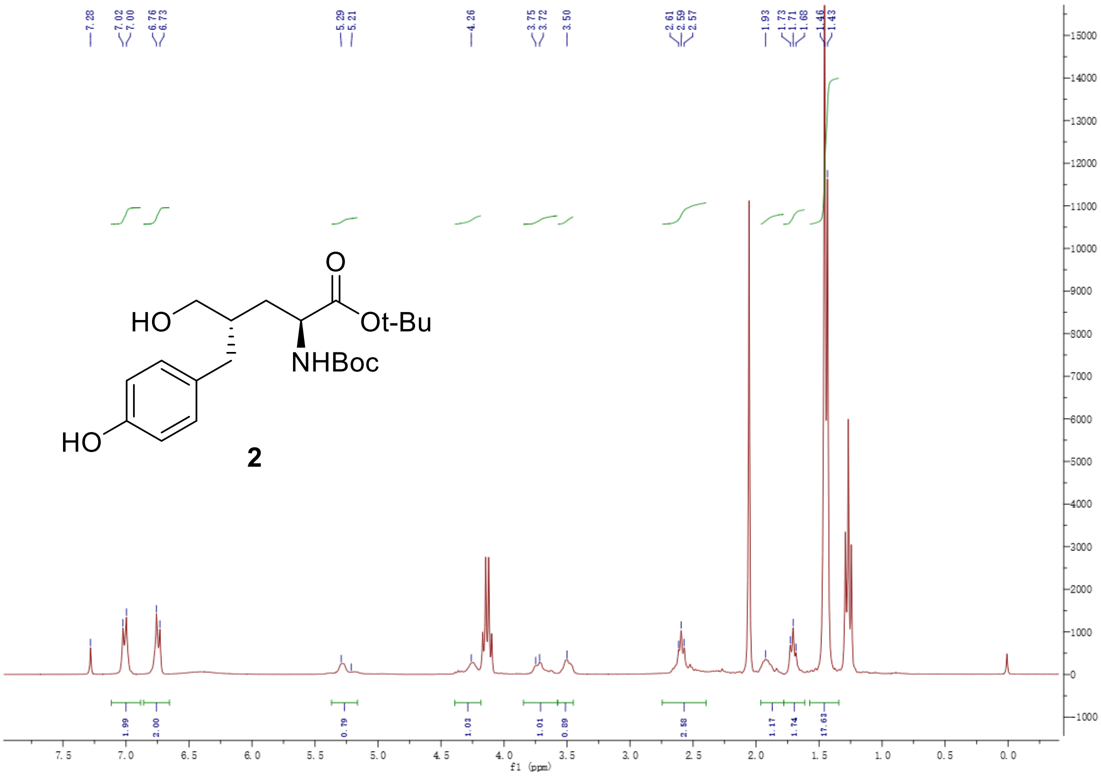
HO
Ot-Bu
NHBoc
HO
2
f1 (ppm)

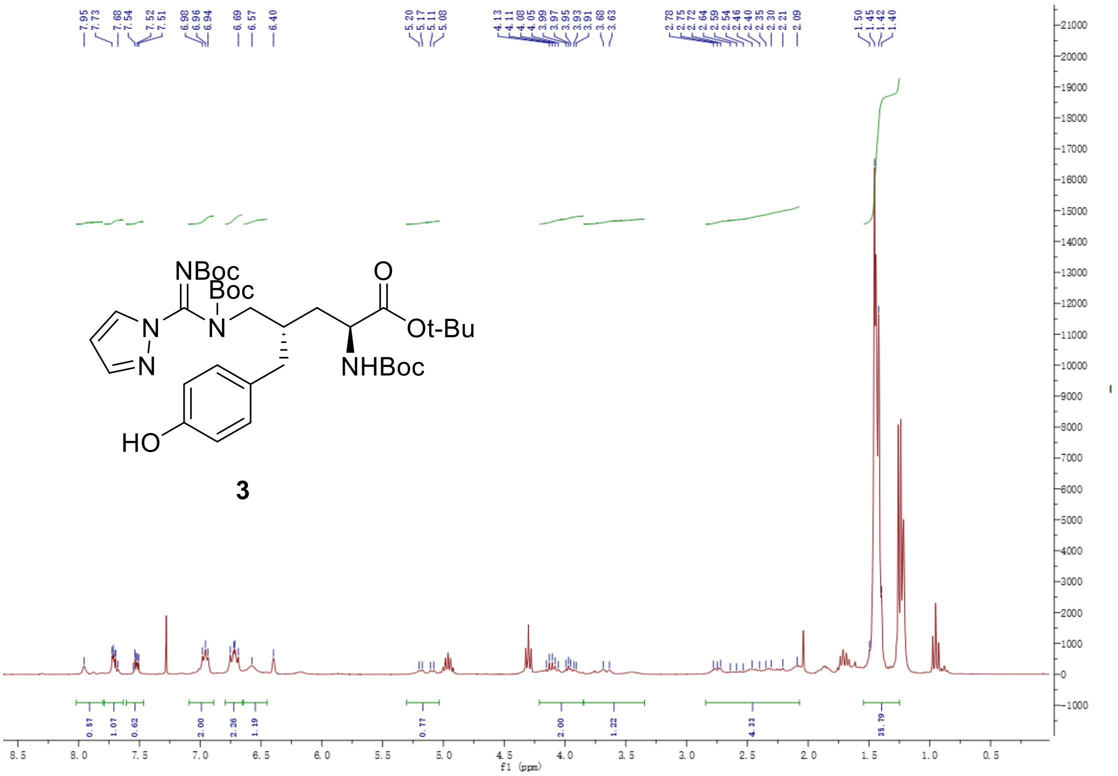
NBoc
Boc
N
N
N
Ot-Bu
NHBoc
HO
3
f1 (ppm)

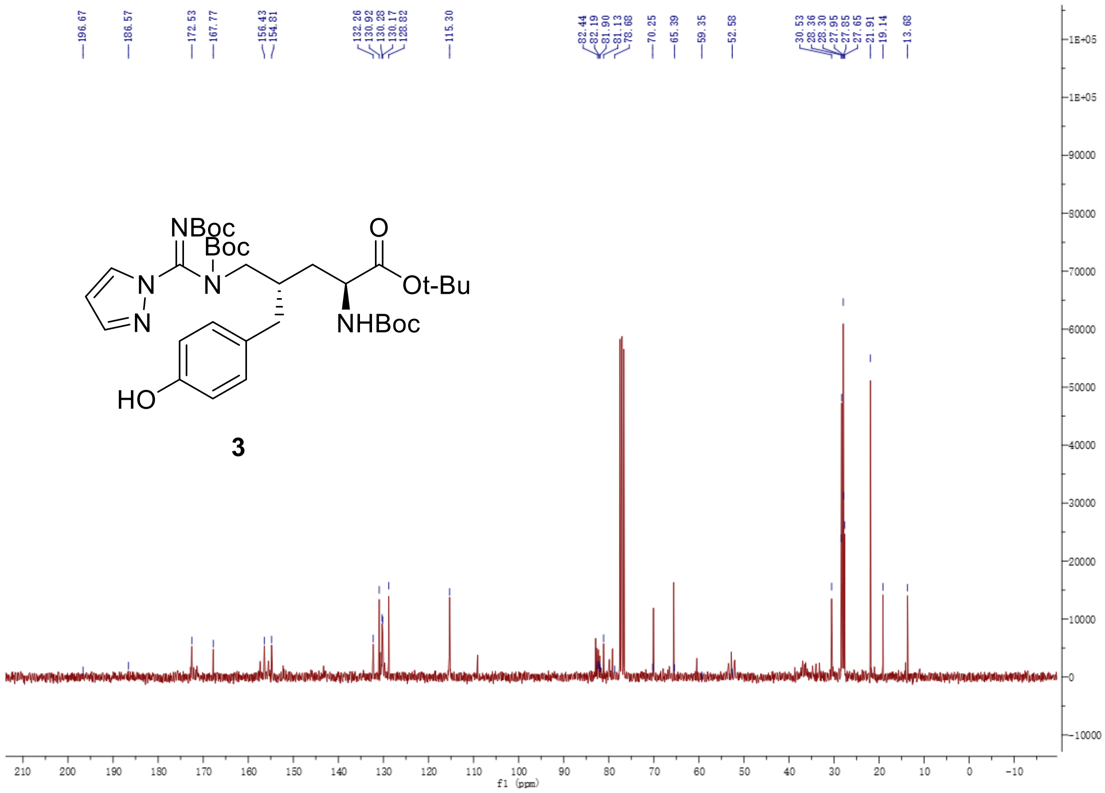
196.67
186.57
172.53
167.77
156.43
154.81
132.26
130.92
130.28
130.17
128.82
115.30
82.44
82.19
81.90
81.13
78.68
70.25
65.39
59.35
52.58
30.53
28.36
28.30
27.95
27.85
27.65
21.91
19.14
13.68
NBoc
Boc
N
N
N
O
Ot-Bu
NHBoc
HO
3
f1 (ppm)

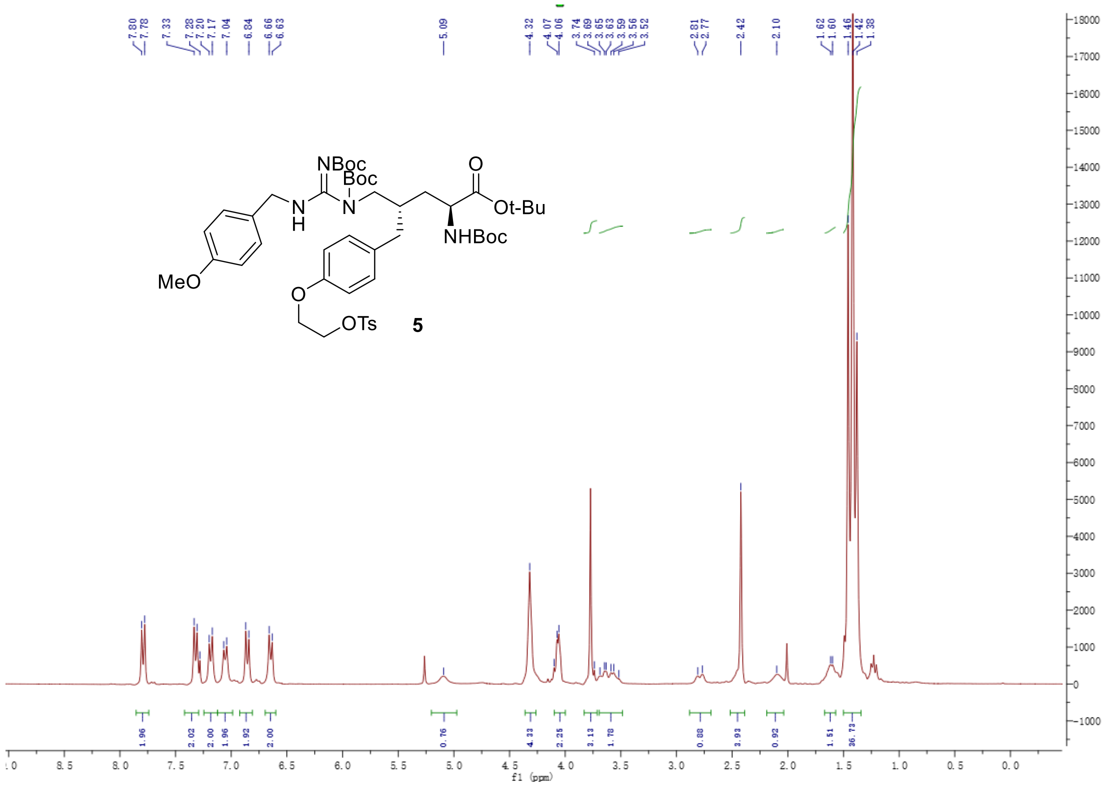
7.80
7.78
7.33
7.28
7.20
7.17
7.04
6.84
6.66
6.63
5.09
4.32
4.07
4.06
3.74
3.69
3.65
3.63
3.59
3.56
3.52
2.81
2.77
2.42
2.10
1.62
1.60
1.46
1.42
1.38
NBoc
Boc
N
H
N
O
Ot-Bu
NHBoc
MeO
O
OTs
5
1.96
2.02
2.00
1.96
1.92
2.00
0.76
4.33
2.25
3.12
1.78
0.88
3.93
0.92
1.51
36.73
f1 (ppm)

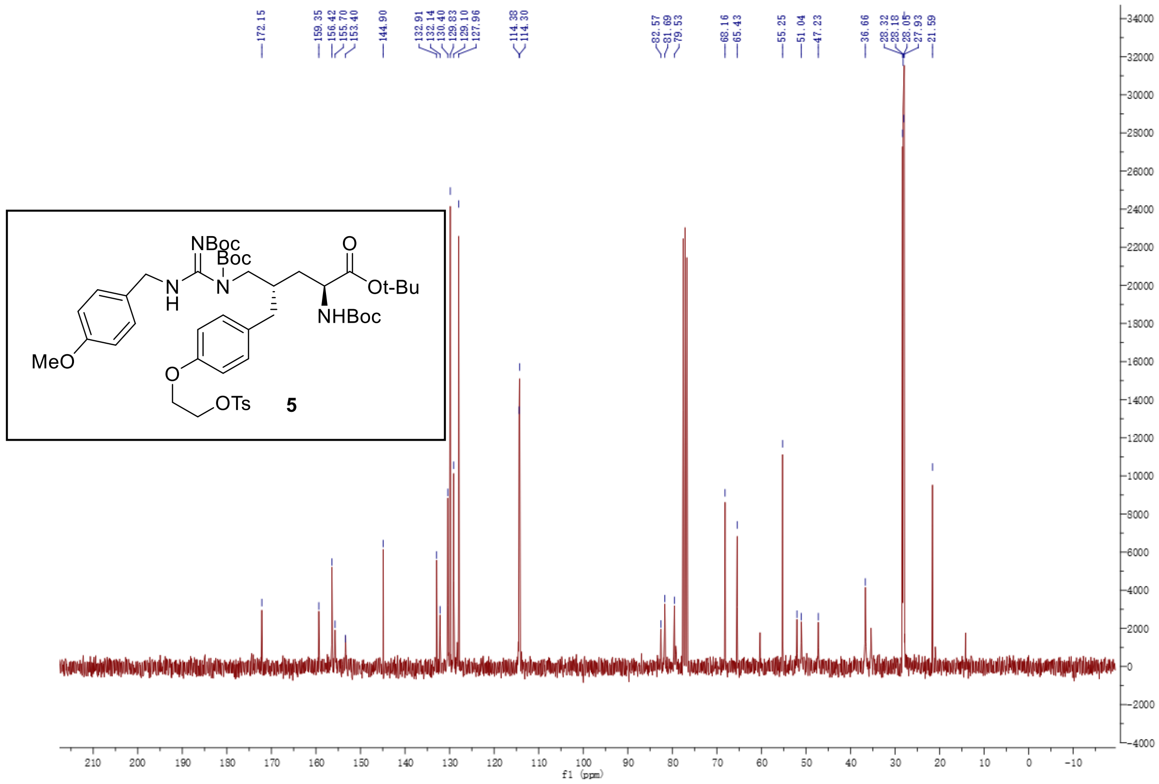

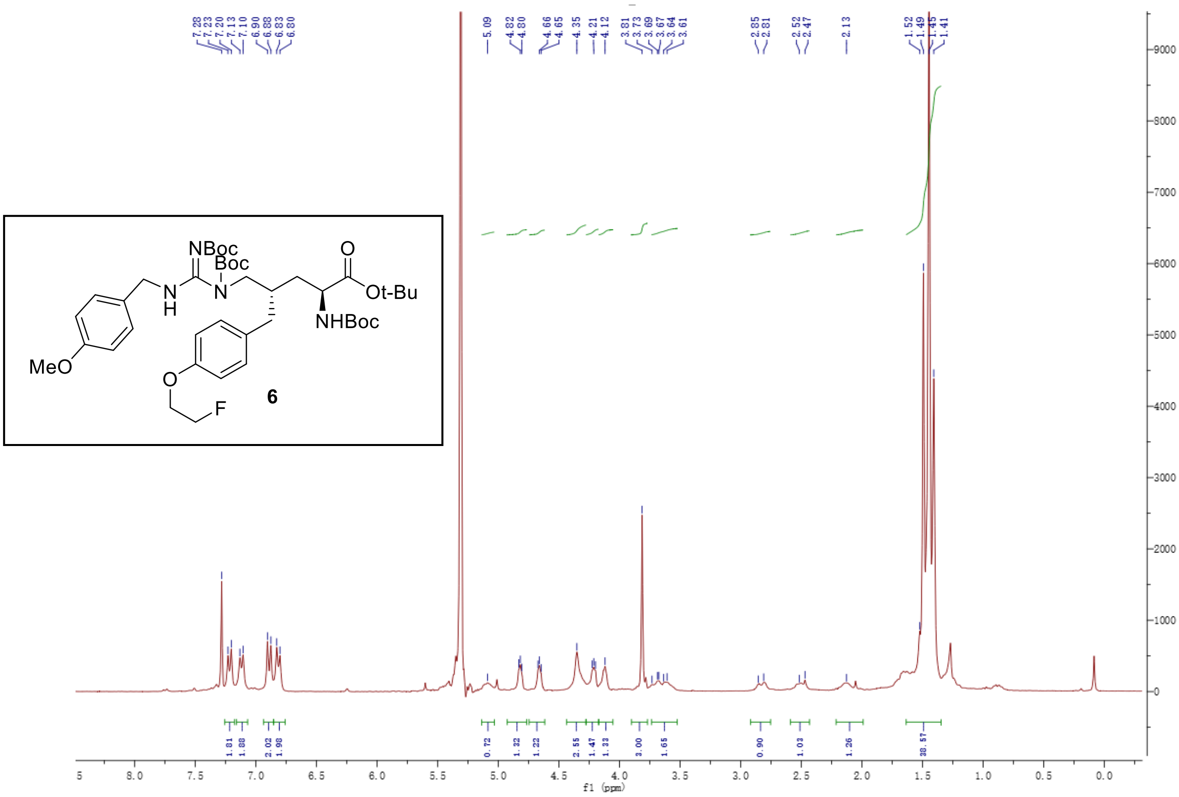

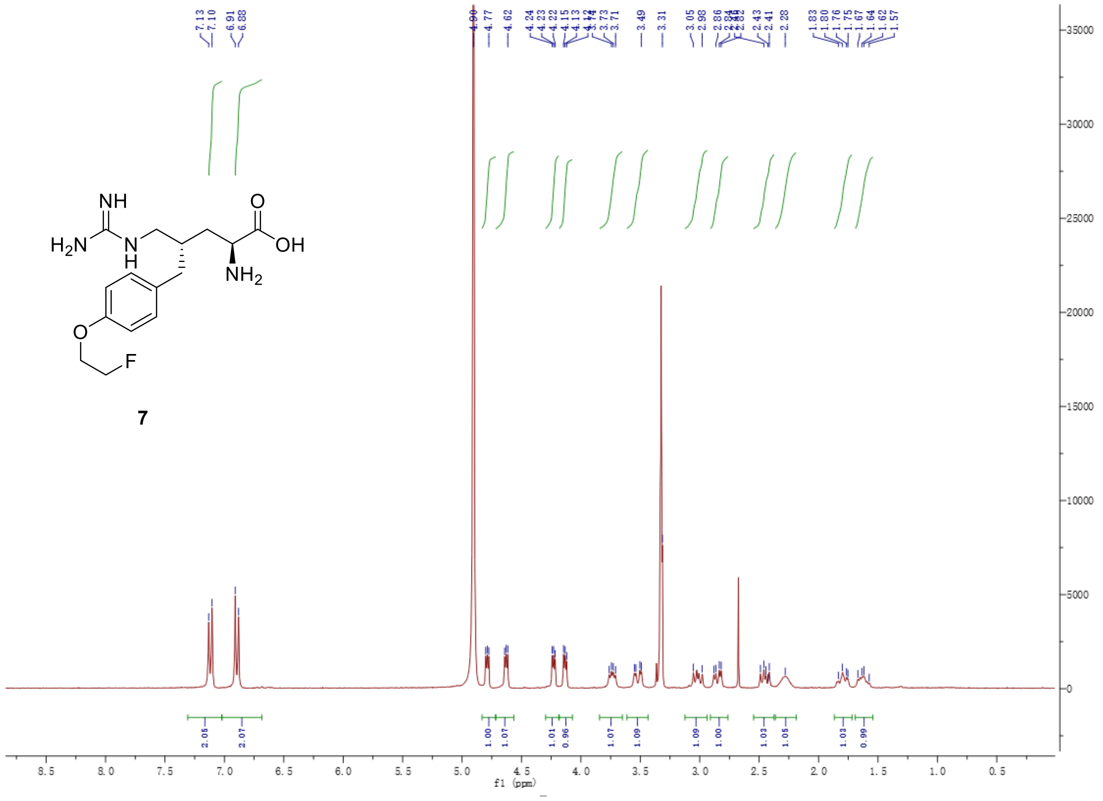
NH
O
H_2N
N
H
OH
NH_2
O
F
7
8.5 8.0 7.5 7.0 6.5 6.0 5.5 5.0 4.5 4.0 3.5 3.0 2.5 2.0 1.5 1.0 0.5
f1 (ppm)
35000 30000 25000 20000 15000 10000 5000 0

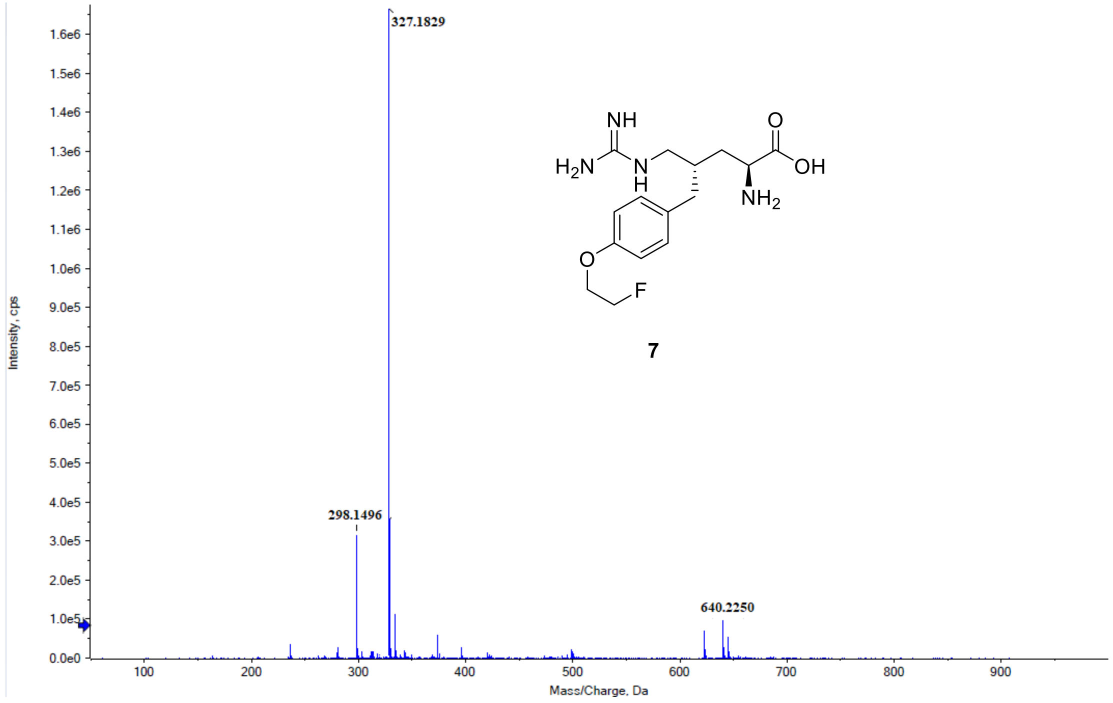
327.1829
298.1496
640.2250
NH
O
H_2N
N
H
OH
NH_2
O
F
7
Intensity, cps
Mass/Charge, Da
